# Resveratrol Possesses Protective Effects in a Pristane-Induced Lupus Mouse Model

**DOI:** 10.1371/journal.pone.0114792

**Published:** 2014-12-11

**Authors:** Zhuo-Long Wang, Xiao-Fang Luo, Meng-Tao Li, Dong Xu, Shuang Zhou, Hou-Zao Chen, Na Gao, Zhen Chen, Ling-Ling Zhang, Xiao-Feng Zeng

**Affiliations:** 1 Department of Rheumatology, Peking Union Medical College Hospital, Peking Union Medical College and Chinese Academy of Medical Sciences, Key Laboratory of Rheumatology and Clinical Immunology, Ministry of Education, Beijing, China; 2 National Laboratory of Medical Molecular Biology, Institute of Basic Medical Science, Peking Union Medical College and Chinese Academy of Medical Sciences, Beijing, China; Xavier Bichat Medical School, INSERM-CNRS - Université Paris Diderot, France

## Abstract

**Background:**

Systemic lupus erythematosus (SLE) is a multisystemic autoimmune disease characterized by the production of autoantibodies. To date, no therapy has been found to satisfactorily treat SLE. SIRT1 deficiency results in the development of an autoimmune syndrome in mice, including a high titer of anti-nuclear antibody in serum, immunoglobulin deposition in the kidney, and immune complex glomerulonephritis. Resveratrol is an activator of SIRT1 and possesses anti-inflammation and immune-regulatory properties.

**Objective:**

To evaluate the preventative effects of resveratrol on a pristane-induced lupus animal model and assess its putative immune modulation effects.

**Methods:**

BALB/c mice received a single intraperitoneal injection of 0.5 ml of pristane on day 1 and then various doses of resveratrol were given to the mice daily starting on day 2 and continuing for seven months. The autoantibodies in serum and supernatants were measured. Single cells isolated from spleen, isolated CD4+ T cells, and CD19+ B cells were cultured with or without resveratrol *in vitro* and assessed by flow cytometry.

**Results:**

Resveratrol attenuated proteinuria, immunoglobuin depositon in kidney, and glomerulonephritis as well as IgG1 and IgG2a in serum in pristane-induced lupus mice. Resveratrol also suppressed CD69 and CD71 expression on CD4+ T cells as well as CD4+ T cell proliferation, induced CD4+ T cell apoptosis, and decreased CD4 IFNγ^+^ Th1 cells and the ratio of Th1/Th2 cells *in vitro*. *In vitro* antibody production and proliferation of B cells were also inhibited.

**Conclusion:**

Resveratrol possesses protective effects in pristane-induced lupus mice and may represent a novel approach for the management of SLE.

## Introduction

Systemic lupus erythematosus (SLE) is a multisystemic autoimmune disease characterized by autoantibodies to components of the cell nucleus. Pathogenic autoantibodies are the primary cause of tissue damage in patients with SLE. Development of the disease is thought to arise due to genetic factors together with environmental triggers [1], [2], [3]. In addition, renal damage is the most important predictor of mortality [4]. However, despite intensive research, no therapy to date has been found to cure SLE, and those that adequately treat the disease may have severe and unfavorable side effects.

Several SLE animal models have been established and play an important role in investigating the mechanisms of the disease. One model is the pristane-induced lupus mouse model, which develops several autoantibodies and immune-complex glomerulonephritis [5], [6]. Studies have shown that these mice have disparate T cell requirements of two subsets of lupus-specific autoantibodies as well as the toll-like receptor 7 (TLR7)-dependent and FcγR-independent production of type I interferon [7], [8]. TLR7 is required for the production of autoantibodies and the development of murine lupus nephritis [9].

Several different elements of the immune system are potential targets for therapeutic intervention in patients with SLE [1], [10], [11]. Current therapeutics used to treat SLE, including glucocorticoids and cyclophosphamide (CTX), are directed at suppressing humoral immunity and the production of autoantibodies as well as helper T cells (Th) and B lymphocytes. In addition, a new generation of biological agents is currently under development; however, the long-term beneficial and adverse effects of such agents remain unknown [10], [11]. Therefore, more effective drugs with a favorable safety profile are urgently needed. Many natural compounds possess immune-modulatory effects and have the potential for treating autoimmune diseases, such as SLE. In this study, we assessed the efficacy of resveratrol for treating SLE.

Resveratrol (3,5,4-trihydroxystilbene) is a natural antimicrobial compound found in various plants and fruits [12], [13]. It has attracted great attention since the discovery of its cardioprotective properties several years ago [13], [14], and the compound has been shown to also possess anti-inflammatory, immune-regulatory, antioxidant, and blood fat-regulatory properties [13], [15]. Moreover, studies have shown that resveratrol can inhibit several experimental autoimmune diseases, including collagen-induced arthritis, encephalomyelitis, colitis, and diabetes, though the mechanisms are not fully understood [16], [17], [18], [19], [20].

Resveratrol is an activator of silent mating type information regulation 2 homolog 1 (SIRT1), which is a class III histone deacetylase [13]. SIRT1 deficiency results in the development of an autoimmune syndrome in mice that manifests as a high titer of anti-nuclear antibody in serum, immunoglobulin deposition in the kidney, and immune complex glomerulonephritis [21], [22].

It has been shown that resveratrol may modualate inflammatory genes and signaling transcription factors, including STAT3, NF-kB, AP-1, and Cyclooxygenase 2 (COX2), which play critical roles in SLE pathogenesis [13]. However, to date, the effects of resveratrol on SLE and pristane-induced lupus have not been explored. Therefore, in this study we evaluated whether resveratrol can prevent the development of pristane-induced lupus in a mouse model.

## Materials and Methods

### Mice

Female BALB/c mice that were 9-10-weeks-old were obtained from Weitonglihua, Ltd. (Beijing, China). All mice were housed at Peking University on a diurnal 12 h light/dark cycle. All experimental protocols described in this study were approved by the Ethical Committee for Animal Experimentation of Peking Union Medical College Hospital.

### Induction and treatment of pristane-induced lupus in mice

Forty female BALB/c mice were randomly divided into the following four groups: (1) Resveratrol A group: 10 BALB/c mice received a single intraperitoneal (i.p.) injection of 0.5 ml of pristane (Sigma-Aldrich, USA) on day 1 and were fed with resveratrol (Sigma-Aldrich, USA) (50 mg/kg/d) daily starting on day 2 for seven months; (2) Resveratrol B group: 10 BALB/c mice received a single i.p. injection of 0.5 ml of pristane on day 1 and were fed with resveratrol (75 mg/kg/d) daily starting on day 2 for seven months; (3) Model control group: 10 BALB/c mice received a single i.p. injection of 0.5 ml of pristane on day 1 but were not given resveratrol; and (4) Normal control group: 10 BALB/c mice not injected with pristane and not fed with resveratrol.

### Proteinuria

Proteinuria was measured on a 0−5+ scale using a colormetric assay strip for albumin (Gaoerbao limited, Guangzhou, China). The following scale was used for assessment: 0 =  absent; 1+ = 10 mg/100 ml; 2+ = 30 mg/100 ml; 3+ = 100 mg/100 ml; 4+ = 300 mg/100 ml; and 5+≥2000 mg/100 ml.

### ELISA

Antibody concentrations were determined by ELISA. The anti-nuclear antibody (ANA), anti-ds-DNA, and anti-RNP/Sm antibodies were diluted 1∶100 in murine serum. (ANA ELISA, Anti-ds-DNA ELISA, and Anti-nRNP/Sm ELISA, respectively; Euroimmun Medizinische Labordiagnosika AG, Germany). For ANA, the secondary antibodies were goat polyclonal anti-mouse IgG-Fc conjugated to horseradish peroxidase (HRP) (Abcam, USA) and goat polyclonal anti-mouse IgM-mu chain conjugated to HRP (Abcam, USA). For the anti-ds-DNA and anti-RNP/Sm antibodies, the secondary antibody was a goat polyclonal anti-mouse IgG-Fc conjugated to HRP (Abcam, USA). Assessment of supernatants and the determination of serum Ig isotypes were performed using the Mouse Immunoglobulin Isotyping ELISA Kit (BD Biosciences, USA). IFN-α were determined using the mouse IFN-alpha ELISA Kit (eBioscience, USA).

### Cell culture and cell sorting

Mice were euthanized 7 months after receiving the single pristane injection and spleens were removed for analysis. CD4+ T cells and CD19+ B cells from splenic mononuclear cells (SMC) were purified by positive selection (Miltenyi Biotec, Bergisch Gladbach, Germany). Cells were cultured in RPMI1640 medium supplemented with 10% fetal bovine serum (FBS), 2 mM L-glutamine, and 100 U/mL penicillin-streptomycin. Cells were cultured at 37°C in a 5% CO_2_ incubator for the indicated time periods. SMCs and CD4+ T cells were activated with ConA (Sigma-Aldrich, USA), anti-CD3 (BD Biosciences PharMingen, USA), or anti-CD3/CD28 (BD Pharmingen, USA) with or without resveratrol (0, 10, 20, 40, or 80 µM) for the indicated time points. CD19+ B cells were activated with lipopolysaccharide (LPS) with or without resveratrol (0, 10, 20, 40, or 80 µM).

### CD69 and CD71

SMCs from pristane-induced lupus mice were activated with ConA (2.5 µg/ml), anti-CD3 (1 µg/ml), or anti-CD3/CD28 (1 µg/ml) with or without resveratrol for 6–12 h (CD69) or 36–48 h (CD71) *ex vivo*. The cells were then stained with rat anti-CD4-PerCP (BD Biosciences PharMingen, USA) and hamster anti-CD69-FITC (BD Biosciences PharMingen, USA) or rat anti-CD71-FITC (BD Biosciences, USA) to investigate the activation of CD4+ T cells by flow cytometry (FACS Aria, BD Biosciences, USA).

### Proliferation assay

Freshly islated SMCs, CD4+ T lymphocytes, and CD19+ B lymphocytes were labeled with Carboxyfluorescein Succinimidyl Ester (CFSE) (CFSE cell proliferation kit, Invitrogen, USA) and cultured under various conditions. Four days later, the cells were analyzed by fow cytometry.

### Apoptosis assay

Freshly isolated CD4+ T cells from the SMC fraction were cultured with or without resveratrol for 36 h *in vitro*. The cells were then stained with Annexin V/propidium iodide (PI) (Alexa Fluor 488 Annexin V and PI kit For Flow Cytometry, respectively; Invitrogen, USA) to evaluate CD4+ T cell apoptosis by flow cytometry.

### Analysis of the helper T cell subpopulation

Freshly isolated SMCs were activated with phorbol-12-myristate-13-acetate (PMA, Sigma-Aldrich, USA) and ionomycine (Sigma-Aldrich, USA) after having been cultured with or without resveratrol for 2 h. The cells were then stained with anti-CD4-PerCP, anti-IL-4-PE (BD Biosciences, USA), anti-IFN-γ-APC (BD Biosciences, USA), and anti-IL-17-Alexa Fluor 488 (BD Biosciences, USA) to investigate Th1, Th2, and Th17+ cell populations by flow cytometry.

### Histology

One kidney from each animal was fixed in 10% neutral-buffered formalin, cut into 5 µm-thick sections, and then stained with hematoxylin and eosin (H&E). Glomerular cellularity was evaluated by counting the number of nuclei per glomerular cross-section(30–40 glomerular cross-sections per mouse) after staining with hematoxylin and eosin. The other kidney from each animal was embedded in ornithine carbamoyltransferase and cut into 5-µm-thick sections for detecting IgG and IgM by direct immunofluorescence.

### Detection of IgG and IgM in renal tissue by immunofluorescence

Freshly frozen sections (5 µm thick) were stained for IgG and IgM with goat polyclonal antibody anti-mouse IgG-H&L-FITC (1∶80 dilution; Abcam, USA) or goat polyclonal anti-mouse IgM-H&L-FITC (1∶80 dilution; Abcam, USA) antibody. Staining specificity was assessed using standard laboratory blocking and absorption procedures. No cross-reactivity of complement, fibrinogen, or glomerular tissues from controls was observed. The immunoglobuin deposition in kidney was scored as 0, 1+, 2+, 3+, or 4+.

### Statistical analysis

Clinical scores were analyzed using the non-parametric Mann-Whitney U test and histological scores were assessed using the one-way analysis of variance (ANOVA) test. Other data were compared using the Student's t-test or one-way ANOVA. The statistical significance of the various tests was examined by 2-sided hypothesis testing. P values <0.05 were considered statistically significant. Statistical analysis was performed using SPSS version 17.0 (SPSS, Chicago, IL, USA).

## Results

### 1. Protective effect of resveratrol in a pristane-induced lupus animal model

#### 1.1 Proteinuria

At the end of observation period, urinalysis revealed that the proteinuria of the normal control group was 0 or 1+, and 1+ to 3+ for the model control mice. The resveratrol A and resveratrol B groups had a lower proteinuria level compared to the model control group (Z = 2.013; P = 0.044; and Z = 2.071; P = 0.038, respectively; S1 Table). Therefore, resveratrol can prevent the development of proteinuria in this pristane-induced lupus model.

#### 1.2 Histological pathogenesis

**Figure 1:**
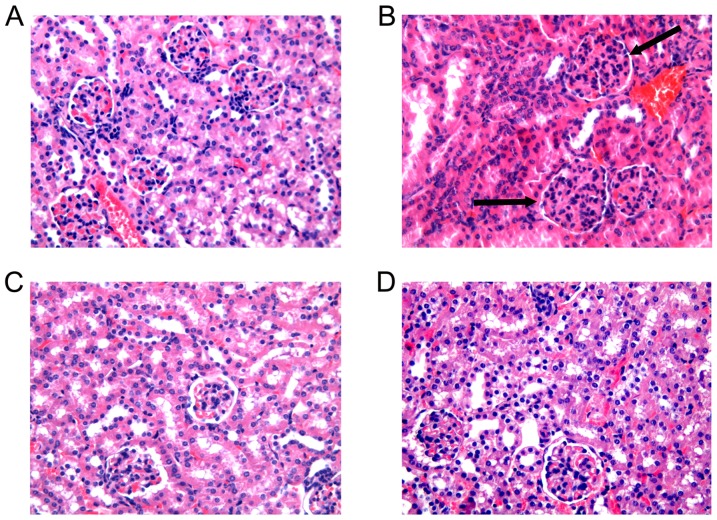
Kidney pathology. The kidney sections were stained with hematoxylin and eosin (H&E) and representative images are shown. (A): Normal glomerulus from a normal control mouse (n = 10). (B): Proliferative glomerular lesions and mononuclear cell infiltration in glomerulus from a model control mouse (n  =  10). (C): A marked reduction in pathological lesions in glomerulus from a mouse from the resveratrol A group (n  =  10) (D): A marked reduction in pathological lesions is observed in glomerulus from a representative resveratrol B mouse (n  =  10). (original magnification×200)

At the end of observation period, light microscopy of kidney sections revealed that 6 mice developed segmental or diffuse proliferative glomerular lesions with mononuclear cell infiltration among 10 model control mice (Fig. 1B): 3 mice among 10 resveratrol A group mice and 10 resveratrol B group mice, respectively. No mouse developed glomerular lesions among the normal control group mice. The mice from the resveratrol A group had a marked reduction in pathological lesions of the glomerulus compared to mice of the model control group (Fig. 1C) and slightly less lymphocyte infiltration was also observed. Similar results were detected in mice from the resveratrol B group as well (Fig. 1D).

The number of nuclei per glomerular cross-section, which is a measure of glomerular cellularity, was 39.3±4.1 for mice in the normal control group. This value was decreased for mice in the resveratrol A and B groups compared to model control mice (44.1±11.4 vs. 52.1±14.6, t = 2.282, p = 0.027; and 42.8±9.9 vs. 52.1±14.6, t = 2.510,p = 0.017, respectively). In contrast, there was no difference in glomerular cellularity between mice in the resveratrol A and B groups (44.1±11.4 vs. 42.8±9.9, respectively; t = 0.441, p = 0.661).

#### 1.3 Immunoglobuin G (IgG) and IgM deposition in kidney

**Figure 2:**
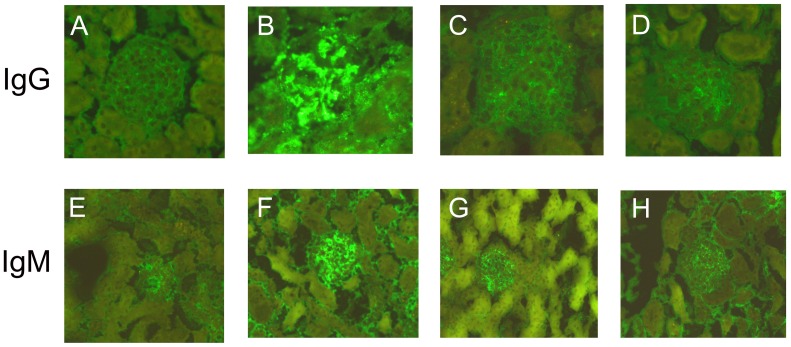
Immunofluorescence microscopy. Glomerulus from a normal control mouse (A, E), model control mouse (B, F), resveratrol A mouse (C, G), and resveratrol B mouse (D, H). Panels A, B, C, and D were stained with goat polyclonal anti-mouse IgG-FITC and panels E, F, G, and H were stained with goat polyclonal anti-mouse IgM-FITC.

At the end of the observation period, IgG deposition in kidney of normal control mice was negative, 0−2+ in model control mice, and 0−1+ in mice from the resveratrol A and B groups (Fig. 2A-D, S2 Table). Moreover, the extent of IgG deposition in the kidney of resveratrol A and B mice was significantly lower than that in the model control mice (Z = 2.294; p = 0.022; and Z = 2.294; p = 0.022, respectively; S2 Table). However, the IgG deposition in mice from the resveratrol A and B groups was not significantly different (P>0.05). Similar results were observed for IgM deposition (Fig. 2E-H; S3 Table). Taken together, these results indicate that resveratrol attenuates pristane-induced nephritis.

### 2. Resveratrol inhibits IgG1 and IgG2α in pristane-induced lupus

**Figure 3:**
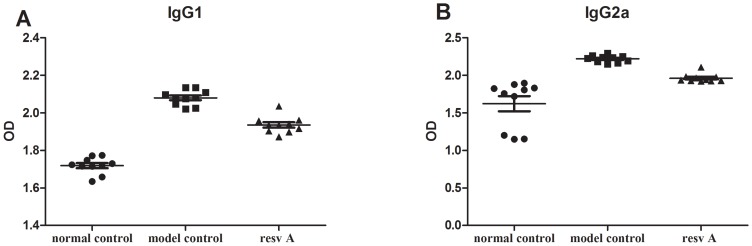
Comparison of serum immunoglobulins at the end of the observation period. (A): IgG1 levels in mice of the resveratrol A group were significantly lower than those in the control group (n = 10 in each group). (B): Reduction of IgG2a levels in mice of the resveratrol A group (n = 10 in each group).

To assess the immune mechanisms responsible for the resveratrol-mediated prevention of ongoing pristane-induced lupus, we assessed its role in the regulation of humoral immunity by measuring total immunoglobulin levels and ANA as well as anti-ds-DNA and anti-RNP/Sm antibody levels in serum. At the end of the observation period, the IgG1 levels in mice from the resveratrol A group were significantly lower than those in the model control group (2.08±0.04 vs. 1.93±0.04, respectively; P<0.01; Fig. 3A). Similar findings were observed for IgG2a levels between the two groups (Fig. 3B), but no differences were observed in IgG2b, IgG3, IgM, and IgA levels between the treatment and the model control groups (data not shown). Similar results were also detected in mice from the resveratrol B group (data not shown).

Throughout the observation period, ANA levels in mice from the resveratrol A and resveratrol B groups were lower than those in mice from the model control group, but the difference was not statistically significant (0.74±0.32 vs. 0.57±0.09, P = 0.137; and 0.74±0.32 vs. 0.576±0.23, P = 0.183, respectively, S1 and S2 Figures). Similar changes were also observed for anti-ds-DNA and anti-RNP/Sm antibodies.

### 3. IFN-α levels in serum

In light of the role of IFN-α in SLE, we analyse IFN-α in serum. At the end of observation period, IFN-α levels in mice from the resveratrol A and resveratrol B groups were lower than those in mice from the model control group, but the difference was not statistically significant (0.110±0.011 vs. 0.115±0.001, P = 0.330; and 0.111±0.003 vs. 0.115±0.001, P = 0.291, respectively.)

### 4. Resveratrol suppresses B lymphocyte proliferation and antibody production

**Figure 4:**
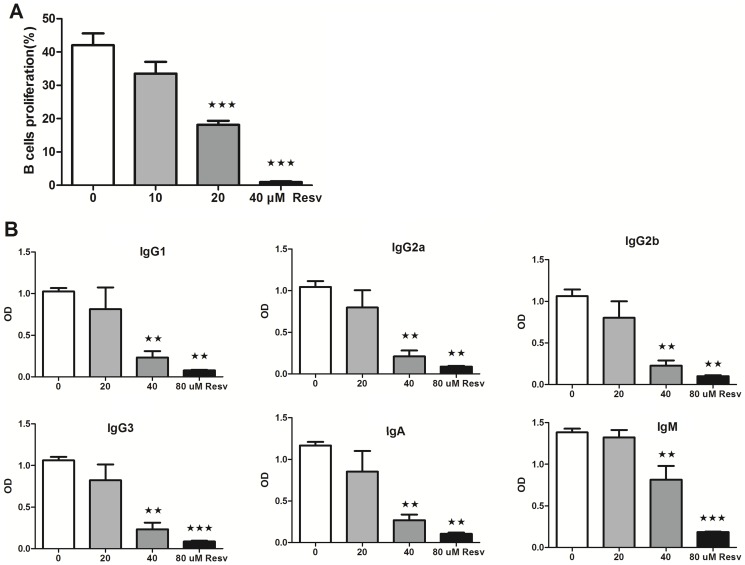
Effects of resveratrol on B lymphocytes *in vitro*. (A): Resveratrol inhibits B lymphocyte proliferation [mean ± standard deviation (SD); n = 8]. (B) Resveratrol decreases antibody production of B lymphocytes. (mean ± SD; n = 3) (★: P<0.05; ★★: <0.01; ★★★: <0.001).

Freshly islated CD19+ B lymphocytes were cultured with LPS (10 µg/ml) and increasing concentrations of resveratrol (0, 10, 20, or 40 µM, respectively) for 4 d *in vitro* and then assessed for proliferation. Resveratrol significantly inhibited B cell proliferation (F = 49.56; p<0.0001) compared to the controls in a concentration-dependent manner (Fig. 4A). To investigate the potential effect of resveratrol on B cells, purified B cells were cultured with LPS (10 µg/ml) with or without resveratrol (0, 20, 40, or 80 µM, respectively) for 7 d *in vitro*, and then Ig isotypes (IgG1, IgG2a, IgG2b, IgG3, IgM, and IgA) were measured in the supernatant. Compared to the controls, resveratrol significantly decreased the production of IgG1 (F = 10.923; P = 0.003), IgG2a (F = 16.001; P = 0.001), IgG2b (F = 16.934; P = 0.001), IgG3 (F = 20.167; p<0.0001), IgM (F = 33.541; p<0.0001), and IgA (F = 14.495; P = 0.001) (Fig. 4B).

### 5. Effects of resveratrol on CD4+ T lymphocyte in vitro

#### 5.1 Resveratrol suppresses CD4+ T lymphocyte activation

**Figure 5:**
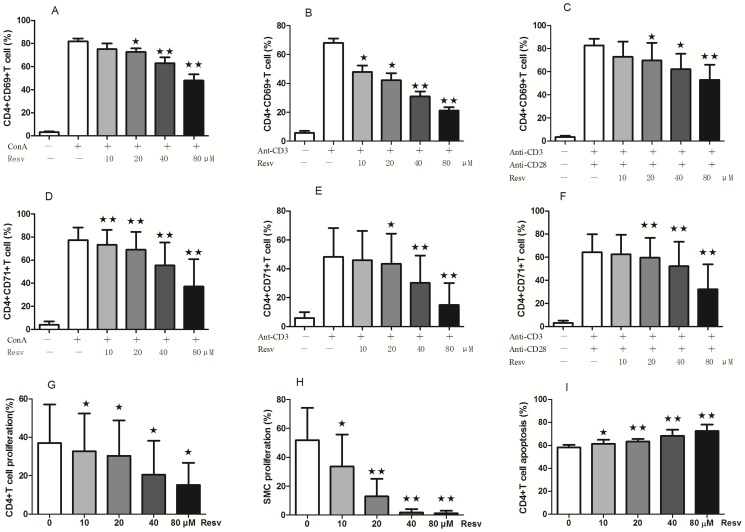
Effects of resveratrol on CD4+ T lymphocytes *in vitro*. Resveratrol suppresses CD69 (A, B, and C) and CD71 (D, E, and F) expression on CD4+ T cells *in vitro* (mean ± SD; A: n = 8; B: n = 6; C: n = 6; D: n = 8; E: n = 8; F: n = 7) Control: SMCs cultured with activator and without resveratrol. (G-H) Resveratrol suppresses cell proliferation. G: CD4+ T cells; H: SMCs. (mean ± SD; n = 6). (I) Resveratrol induces CD4+ T cell apoptosis (mean ± SD; n = 5) (★: P<0.05; ★★: P<0.01).

Freshly isolated SMCs from pristane-induced lupus mice were activated with ConA (2.5 µg/ml) with or without resveratrol and the levels of CD69 and CD71 were then measured, which are markers of CD4+ T lymphocyte activation. The expression level of CD69 on CD4+ T lymphocytes was 3.28±1.33% without activator, but when the cells were incubated with ConA and resveratrol (0, 10, 20, 40, or 80 µM), the levels increased to 81.88±6.98%, 75.04±14.19%, 72.60±9.28%, 62.98±12.02%, and 47.97±15.12%, respectively (Fig. 5A). Therefore, resveratrol signficantly decreased the level of CD69 expression on CD4+ T lymphocytes activated with ConA in a dose-dependent manner. Moreover, a similar effect of resveratrol on CD4+ T lymphocytes was observed when they were activated with anti-CD3 or anti-CD3/CD28 antibodies (Fig. 5B and C, respectively).

The expression level of CD71 on CD4+ T lymphocytes was 3.98±2.92% without activator, but increased to 77.38±10.87% when activated with ConA. However, the addition of 10, 20, 40, or 80 µM resveratrol decreased CD71 expression levels to 73.19±12.94%, 69.05±15.39%, 55.35±19.82%, and 37.13±23.67%, respectively, indicating that resveratrol signficantly decreased the expression level of CD71 on CD4+ T lymphocytes activated with ConA in a dose-dependent manner (Fig. 5D). Similar effects were also observed when the cells were activated with anti-CD3 or anti-CD3/CD28 antibodies (Fig. 5E and F, respectively).

#### 5.2 Resveratrol suppresses CD4+ T lymphocyte and SMC proliferation

Freshly isolated CD4+ T lymphocytes from pristane-induced lupus mice were activated with anti-CD3/CD28 antibodies (3 µg/ml) and interleukin-2 (IL-2) (200 U/ml) with or without resveratrol (0, 10, 20, 40, or 80 µM). We found that cell proliferation decreased after treatment with resveratrol in a dose-dependent manner (Fig. 5G). This dose-dependent reduction was also observed in SMCs activated with ConA (5 µg/ml) and treated with resveratrol compared to untreated control (Fig. 5H).

#### 5.3 Resveratrol induces CD4+ T cell apoptosis

**Figure 6:**
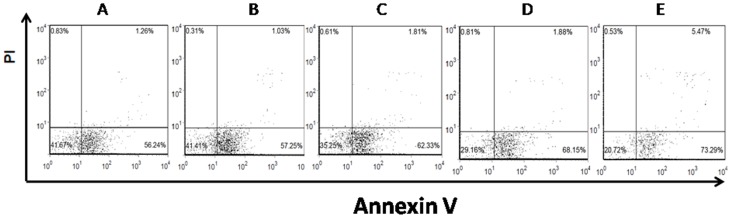
Examples of dot plots showing apoptosis of CD4+ T cells *in vitro*. CD4+ T cells cultured with different concentrations of resveratrol. (A): CD4+ T cells cultured with 0 µM resveratrol. (B): CD4+ T cells cultured with 10 µM resveratrol. (C): CD4+ T cells cultured with 20 µM resveratrol. (D): CD4+ T cells cultured with 40 µM resveratrol. (E) CD4+ T cells cultured with 80 µM resveratrol.

Freshly islated CD4+ T cells were cultured with increasing concentrations of resveratrol for 36 h *in vitro* and then assessed for apoptosis. We found that treatment with 0, 10, 20, 40, or 80 µM resveratrol induced an apoptosis rate of 58.21±2.33%, 61.25±3.68%, 63.45±2.05%, 68.40±5.23%, and 72.56±5.50%, respectively (Fig. 5I and Fig. 6).

#### 5.4 Resveratrol reduces the percentage of Th1 cells and decreases the ratio of Th1/Th2 cells

**Figure 7:**
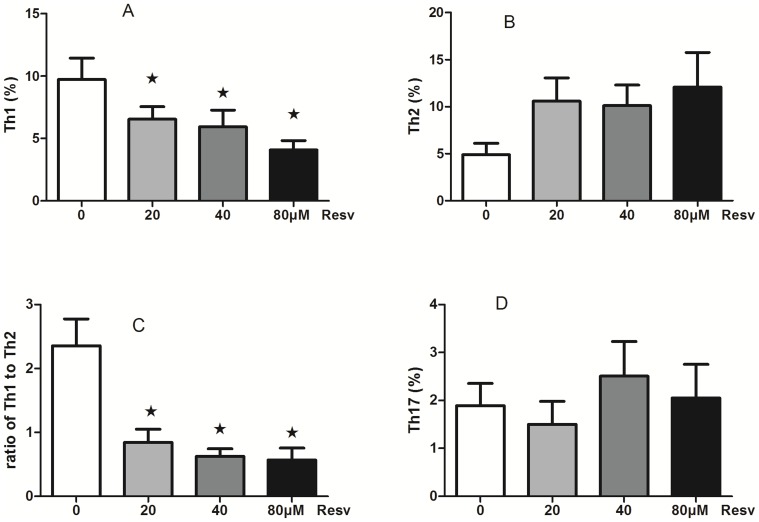
The effect of resveratrol on the subset of T helper cells. (A): Resveratrol inhibits CD4 IFNγ^+^ Th1 cells in a dose-dependent manner. (B): Resveratrol has no effect on CD4 IL-4^+^ Th2 cells. (C): Resveratrol decreases the ratio of Th1/Th2 cells in a dose-dependent manner. (D): Resveratrol had no effect on CD4 IFNγ^−^IL-17^+^ Th17 cells. (mean ± SD; n = 6, ★: P<0.05).

Freshly isolated SMCs were activated with PMA and ionomycine after having been cultured with resveratrol (0, 20, 40, or 80 µM) for 2 h to investigate Th1, Th2, and Th17+ cell populations by flow cytometry. We found that CD4 IFNγ^+^ Th1 cells decreased after treatment with resveratrol in a dose-dependent manner (P<0.05; Fig. 7A), although resveratrol had no effect on CD4 IL-4^+^Th2 cells or CD4 IFNγ^−^IL-17^+^ Th17 cells (Fig. 7B, Fig. 7D, respectively). Importantly, we found that the ratio of Th1/Th2 cells (CD4 IL-4^+^Th2) decreased after treatment with resveratrol in a dose-dependent manner (P<0.05; Fig. 7C),

## Discussion

SLE is an autoimmune disease that responds poorly to current therapy options in many cases. Our study showed for the first time that resveratrol can attenuate proteinuria, decrease IgG and IgM deposition in kidney, and reduce kidney histological lesions, suggesting that resveratrol can effectively prevent pristane-induced lupus in an animal model.

CD4+ T cells and B cells are intimately associated with SLE and pristane-induced lupus [1], [7],[23]. In humoral immunity, helper T cells interact with B lymphocytes and stimulate their proliferation and differentiation, resulting in the production of antibodies to components of the cell nucleus. Our results suggest that resveratrol has a profound inhibitory effect on CD4+ T cells and B cells. Moreover, we showed that resveratrol inhibits the activation of CD4+ T cells *in vitro*, similar to a previous report [24]. Our results also indicate that resveratrol inhibits the proliferation of CD4+ T cells *and* induces cellular apoptosis *in vitro*. More than half of the T cells were already apoptotic (Anexin V+PI−) in the absence of treatment with resveratrol in our study, which is related to the culture duration of up to 36 h *in vitro*. Indeed, it is known that the viability of T cells decreases as culture duration is prolonged[25], [26]. In addition, resveratrol can inhibit the antibody production and proliferation of B cells *in vitro*, which is consistent with previous reports [27].

Taken together, our results suggest that resveratrol has protective properties in a pristane-induced lupus animal model that appear to be attributed to its inhibitory effect on CD4+ T cells and B cells.

The detailed mechanisms responsible for resveratrol-mediated effects remain unclear, though it is postulated that the compound may inhibit CD4+ T cells through effects on SIRT1. Resveratrol is an activator of SIRT1, which is essential for maintaining T cell tolerance through AP-1 in mice [21], [28]. Resveratrol-induced apoptosis may be mediated by Fas, Bcl-2, Bax, p53, or by depolarizing mitochondrial membranes and activating Caspase 9 [29], [30], [31], [32]. Resveratrol induces growth arrest through activation of FOXO transcription factors in prostate cancer cells [33] and suppresses the immune response through CD28/CTLA-4 and CD80 co-stimulatory pathways [34].

Antigen-presenting cells (APCs) present antigens to naive T cells during the recognition phase of immune responses to initiate these responses, and some APCs present antigens to differentiated T cells during the effector phase to trigger elimination of the antigens. Resveratrol can affect dendritic cell maturation and the antigen presentation capacity *in vitro* [35]. However, our results do not rule out a regulatory role of resveratrol in dendritic cells.

Inflammation that is induced by the deposition of antigen-Ab complexes in kidney is an important mechanism for the development of glomerular lesions. Resveratrol inhibits COX expression by suppressing NF-kappa B activation [36] and also inhibits TNF-α-induced inflammation in fibroblasts by activating SIRT1 [37]. The resveratrol-mediated suppression of inflammation may also be benefical for reducing pristane-induced lupus glomerulonephritis.

Imbalance towards Th1 predominance is associated with acceleration of lupus-like autoimmune syndrome in MRL mice [38]. Recent findings in lupus glomerulonephritis show that Th1and Th17 cells have an essential role in the development of diffuse proliferative lupus nephritis and Th2 cytokines have an essential role membranous lupus nephritis [39]. Our study found that CD4 IFNγ^+^ Th1 cells, the ratio of Th1/Th2 cells, B cell, and Th1 cytokine promoting Ig (IgG2a, IgG3) decreased after treatment with resveratrol in a dose-dependent manner. Our findings suggest resveratrol may have effects for alleviating diffuse proliferative lupus nephritis. Resveratrol may also have effects for alleviating membranous lupus nephritis for it supressed Th2 cytokine promoting immunoglobulin (IgG1).

At the end of the observation period, ANA, anti-ds-DNA antibody and IFN-**α** levels in mice from the resveratrol treatment groups were lower than those in mice from the Model control group, but the differences were not statistically significant. The mechanism for this result remains unclear and will require further investigation through a well-designed study (including larger sample numbers) in the future.

## Conclusions

In summary, our findings suggest that resveratrol has protective effects for alleviating pristane-induced lupus in mice and may represent a novel approach for managing SLE. Additional studies in other lupus models and well-designed clinical trials are needed to confirm these findings and explore its therapeutic effects in humans, respectively.

## Supporting Information

S1 Figure Comparison of serum antibodies at the end of the observation period. (A): ANA level in resveratrol treatment group is lower than that in the control group, but there is no significantly statistical difference (n = 10 in each group). (B): Anti-ds-DNA level in resveratrol treatment group is lower than that in the control group, but there is no significantly statistical difference (n = 10 in each group). (C): Anti-RNP/Sm antibody level in resveratrol treatment group is lower than that in the control group, but there is no significantly statistical difference (n = 10 in each group).
(TIF)

S2 Figure **Variation of serum ANA level in different time course.**
(TIF)

S1 Table **Comparison of proteinuria.**
(DOCX)

S2 Table **Comparison of IgG deposition in kidney.**
(DOCX)

S3 Table **Comparison of IgM deposition in kidney.**
(DOCX)
