# Supplementary figures and images for: Resveratrol Possesses Protective Effects in a Pristane-Induced Lupus Mouse Model
Source: PLoS One. 2014 Dec 11;9(12):e114792. doi: 10.1371/journal.pone.0114792 (PMC4263676; doi:10.1371/journal.pone.0114792)

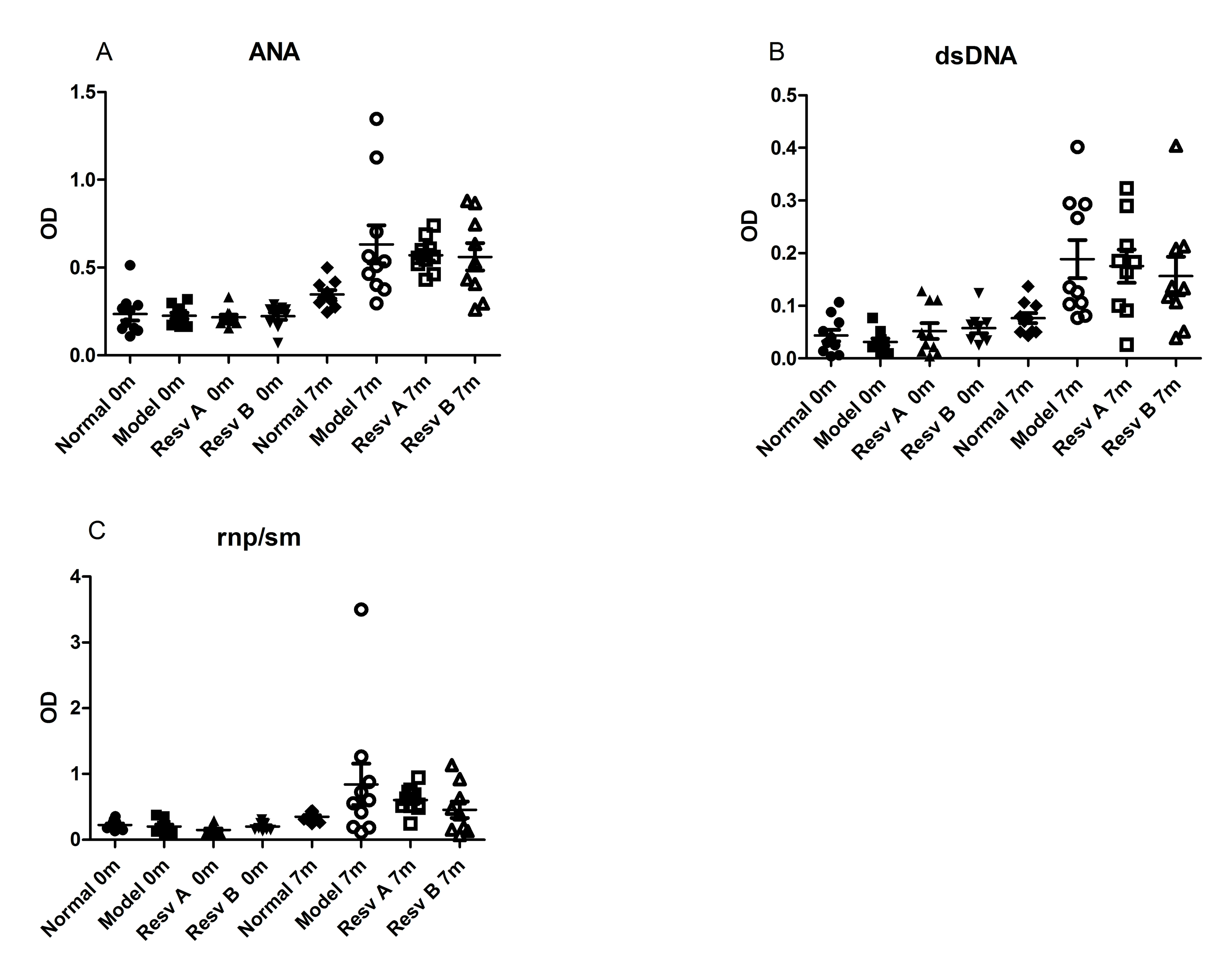

Supplement: S1 Figure — Comparison of serum antibodies at the end of the observation period. (A): ANA level in resveratrol treatment group is lower than that in the control group, but there is no significantly statistical difference (n = 10 in each group). (B): Anti-ds-DNA level in resveratrol treatment group is lower than that in the control group, but there is no significantly statistical difference (n = 10 in each group). (C): Anti-RNP/Sm antibody level in resveratrol treatment group is lower than that in the control group, but there is no significantly statistical difference (n = 10 in each group). (TIF) [file pone.0114792.s001.tif]

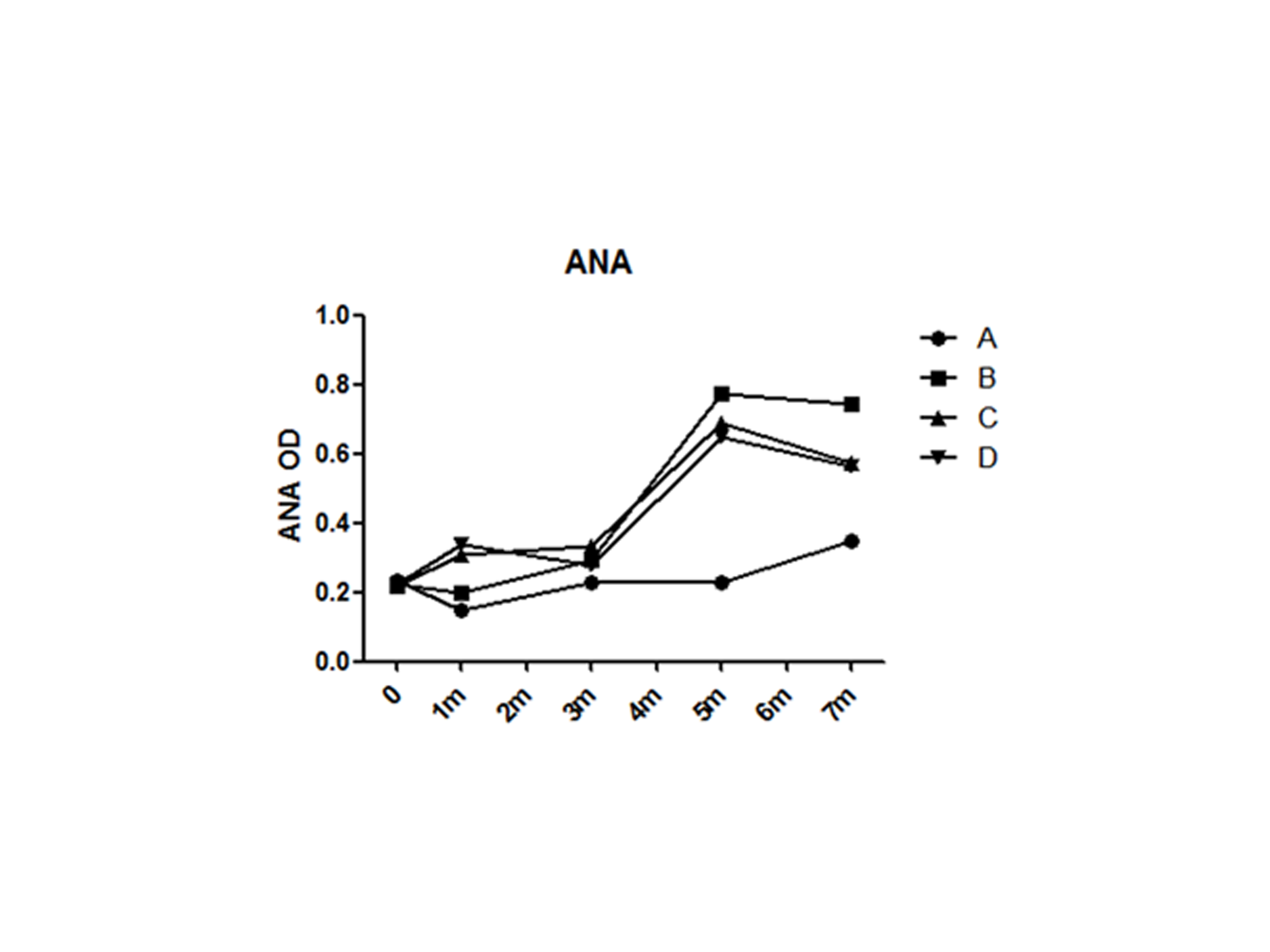

Supplement: S2 Figure — Variation of serum ANA level in different time course. (TIF) [file pone.0114792.s002.tif]
