# Supplementary material for: Resveratrol Possesses Protective Effects in a Pristane-Induced Lupus Mouse Model
Source: PLoS One. 2014 Dec 11;9(12):e114792. doi: 10.1371/journal.pone.0114792 (PMC4263676; doi:10.1371/journal.pone.0114792)
Supplement: S1 Table — Comparison of proteinuria. (DOCX) [file pone.0114792.s003.docx]

Table S1. Comparison of proteinuria

| Group | *N* | *0* | 1+ | 2+ | 3+ | 4+ | 5+ | Z | *P* |
| --- | --- | --- | --- | --- | --- | --- | --- | --- | --- |
| Model control group | 10 | 0 | 2 | 6 | 2 | 0 | 0 |  |  |
| Resveratrol A group | 10 | 0 | 6 | 4 | 0 | 0 | 0 | -2.013 | ＜0.05 |
| Resveratrol B group | 10 | 0 | 7 | 3 | 0 | 0 | 0 | -2.071 | ＜0.05 |
