# Supplementary material for: Resveratrol Possesses Protective Effects in a Pristane-Induced Lupus Mouse Model
Source: PLoS One. 2014 Dec 11;9(12):e114792. doi: 10.1371/journal.pone.0114792 (PMC4263676; doi:10.1371/journal.pone.0114792)
Supplement: S3 Table — Comparison of IgM deposition in kidney. (DOCX) [file pone.0114792.s005.docx]

Table S3. Comparison of IgM deposition in kidney

| Group | *N* | *0* | 1+ | 2+ | 3+ | Z | *P* |
| --- | --- | --- | --- | --- | --- | --- | --- |
| Model control group | 10 | 5 | 4 | 1 | 0 |  |  |
| Resveratrol A group | 10 | 9 | 1 | 0 | 0 | -1.933 | ＜0.05 |
| Resveratrol B group | 10 | 9 | 1 | 0 | 0 | -1.933 | ＜0.05 |
